# Population genetic structuring of methicillin-resistant *Staphylococcus aureus* clone EMRSA-15 within UK reflects patient referral patterns

**DOI:** 10.1099/mgen.0.000113

**Published:** 2017-07-04

**Authors:** Tjibbe Donker, Sandra Reuter, James Scriberras, Rosy Reynolds, Nicholas M. Brown, M. Estée Török, Richard James, East of England Microbiology Research Network, David M. Aanensen, Stephen D. Bentley, Matthew T. G. Holden, Julian Parkhill, Brian G. Spratt, Sharon J. Peacock, Edward J. Feil, Hajo Grundmann

**Affiliations:** ^1^​ Nuffield Department of Medicine, University of Oxford, John Radcliffe Hospital, Headley Way, Oxford OX3 9DU, UK; ^2^​ Department of Medical Microbiology, University Medical Centre Groningen, University of Groningen, Groningen, The Netherlands; ^3^​ Department of Medicine, University of Cambridge, Cambridge, UK; ^4^​ Pathogen Genomics, Wellcome Trust Sanger Institute, Hinxton, UK; ^5^​ The Milner Centre for Evolution, Department of Biology and Biochemistry, University of Bath, Bath, UK; ^6^​ British Society for Antimicrobial Chemotherapy, UK; ^7^​ North Bristol NHS Trust, Bristol, UK; ^8^​ Cambridge University Hospitals NHS Foundation Trust, Cambridge, UK; ^9^​ Public Health England, UK; ^10^​ Department of Physics and Centre for Networks and Collective Behaviour, University of Bath, Bath, UK; ^11^​ East of England Microbiology Research Network, UK; ^12^​ Faculty of Medicine, School of Public Health, Imperial College, London, UK; ^13^​ School of Medicine, University of St Andrews, St Andrews, UK; ^14^​ Department of Infection Prevention and Hospital Hygiene, University Medical Centre Freiburg, Medical Faculty, University of Freiburg, Freiburg, Germany

**Keywords:** antimicrobial Resistance, hospital network, MRSA

## Abstract

Antibiotic resistance forms a serious threat to the health of hospitalised patients, rendering otherwise treatable bacterial infections potentially life-threatening. A thorough understanding of the mechanisms by which resistance spreads between patients in different hospitals is required in order to design effective control strategies. We measured the differences between bacterial populations of 52 hospitals in the United Kingdom and Ireland, using whole-genome sequences from 1085 MRSA clonal complex 22 isolates collected between 1998 and 2012. The genetic differences between bacterial populations were compared with the number of patients transferred between hospitals and their regional structure. The MRSA populations within single hospitals, regions and countries were genetically distinct from the rest of the bacterial population at each of these levels. Hospitals from the same patient referral regions showed more similar MRSA populations, as did hospitals sharing many patients. Furthermore, the bacterial populations from different time-periods within the same hospital were generally more similar to each other than contemporaneous bacterial populations from different hospitals. We conclude that, while a large part of the dispersal and expansion of MRSA takes place among patients seeking care in single hospitals, inter-hospital spread of resistant bacteria is by no means a rare occurrence. Hospitals are exposed to constant introductions of MRSA on a number of levels: (1) most MRSA is received from hospitals that directly transfer large numbers of patients, while (2) fewer introductions happen between regions or (3) across national borders, reflecting lower numbers of transferred patients. A joint coordinated control effort between hospitals, is therefore paramount for the national control of MRSA, antibiotic-resistant bacteria and other hospital-associated pathogens.

## Abbreviations

CC, Clonal Complex; MRSA, methicillin-resistant Staphylococcus aureus; PI, Possible Introduction; SNP, Single Nucleotide Polymorphism; spa, staphylococcal protein A; ST, Sequence Type.

## Data Summary

A full list of accession numbers for the whole-genome sequence data used in this study, as well as the contact matrix describing the numbers of shared patients between hospitals, are available as Supplementary Material.

## Impact Statement

To curb the spread of antibiotic resistance in health care, the spread of resistance between hospitals needs to be understood. Here we test the extent to which patient referrals influence the frequency of inter-hospital transmission. We used whole-genome sequencing to compare methicillin-resistant *Staphylococcus aureus* isolated from patients in hospitals across the UK and Ireland, allowing the comparison of bacterial populations in hospitals with a higher resolution than traditional typing methods. The genetic population structure mirrored the hierarchical structure of the patient referral network, with distinct bacterial populations at each level, from hospitals to regions to countries, confirming a key role of patient referral in the spread of resistant bacteria among hospitals. Coordinated control efforts between hospitals within the same regions are thus vital to mitigate the spread of MRSA and other antibiotic-resistant hospital-associated pathogens between hospitals, because most introductions into a given hospital originate from hospitals that directly transfer large numbers of patients.

## Introduction

The increase in antibiotic resistance represents a global threat to public health in general, and to the health of hospitalised patients in particular. Hospitalised patients are more susceptible to infections, often with opportunistic bacteria that are otherwise harmless commensals. Such infections are usually treatable with antibiotics and pose no serious threat to health, but increasing rates of resistance can make these infections potentially life-threatening. Curbing the spread of resistance includes the more prudent use of available antibiotics to reduce the selective pressure on bacterial populations [1–3] and implementation of effective control strategies to prevent the spread of multidrug-resistant strains to unaffected patient populations. In order to design control strategies, a thorough understanding of the mechanisms by which resistance spreads between patients and bacterial populations in different hospitals is required.

One of the most likely modes of transmission between hospitals is via inter-hospital transfer of colonised patients [4]; each transferred patient offers an opportunity for resistant bacteria to be exchanged from one hospital to the next. The overall population of transferred patients creates connectivity between all health care institutions to form a single health care network [5–7], through which resistant bacteria may spread. The structure of this network influences the spread of resistance, with faster spread towards the tertiary care institutions and within closely connected regions, and slower dispersal between hospitals in other regions [8]. Most evidence for inter-hospital spread of resistant bacteria is anecdotal, based on observed cases that signified the index case of a hospital outbreak [9, 10]. Although the identification of these introductions is important, such evidence has rarely been considered within the broader context of sustained transmission between hospitals by transferred patients on a national level.

Methicillin-resistant *Staphylococcus aureus* (MRSA) is often used as a model organism to study the spread of drug-resistant bacteria, owing both its public health importance and favourable aspects of its population biology that make it possible to track individual clones over time and space [11, 12]. Low rates of recombination help to maintain relatively stable patterns of spatio-temporal variation [13]. In the United Kingdom, two predominant MRSA clones, EMRSA-15 [Clonal Complex (CC) 22] and EMRSA-16 (CC 30), have successfully spread through hospitals since the early 1990s [14], with the latter known to have first emerged in the English midlands in the mid-1980s [15]. The resulting nationwide epidemic of MRSA infections in the 1990’s prompted the introduction of a raft of control policies in the UK, including mandatory surveillance of MRSA bacteraemia [16]. Over the past decade MRSA bacteraemia rates have dropped substantially [16–18], apparently indicating that MRSA bacteraemias can be prevented by improved infection control practices in individual hospitals. However, it is still unclear how these epidemic MRSA clones were able to spread to all UK hospitals over such a short period and what forces attenuated their epidemic behaviour.

A number of studies have shed light on the local processes underlying the dispersal of MRSA, in particular between neighbouring hospitals. These showed that hospitals that exchanged large numbers of patients also harboured MRSA populations with similar spa types [19] and an increased proportion of genetically nearly identical isolates within a single clonal complex [20], indicating an effect of patient transfers on genetic population structure of MRSA. However, the structure of the patient referral networks extends further than the direct connections between neighbouring hospitals, and these higher levels of organisation will inevitably affect the dispersal of MRSA and other antibiotic-resistant bacteria. Moreover, these studies were based on the community-acquired clone USA300 (ST8), which confers a significant public health burden in North America. This clone is not restricted to health-care settings, and therefore is likely to exhibit epidemiological properties that are quite distinct from those of the two hospital-adapted clones, EMRSA-15 and EMRSA-16, common in the UK.

In order to obtain a comprehensive view of how the patient referral network affects the dispersal of antibiotic-resistant bacteria, we studied a single MRSA clonal complex, CC22, isolated in hospitals in the United Kingdom and the Republic of Ireland (UK and I). Using whole-genome sequencing, we compared the genetic composition of hospital MRSA populations to determine their relatedness at the highest resolution possible and ascertained the effect of the structure of the patient referral network on the spread of MRSA. Whilst an overview of the genetic structuring of EMRSA-15 in the UK has previously been reported [21] that study focussed more on contextualising local outbreaks than on addressing the role of patient referral in inter-hospital transmission.

## Methods

### Isolate collection

We collected 783 MRSA clonal complex (CC) 22 isolates between 2001 and 2010 from 45 hospitals across the UK and I through the British Society of Antimicrobial Chemotherapy (BSAC) bacteraemia resistance surveillance programme [22, 23]. Additional MRSA CC22 bacteraemia isolates were retrospectively collected from nine hospitals in the East of England between 1998 and 2012. One hospital contributed to both the BSAC collection and the East of England collection, and only non-duplicate isolates were included in the analysis. Hospital names were coded by letters according to their region, to preserve anonymity.

DNA was extracted for each MRSA isolate using a QIAxtractor (QIAGEN), and was prepared for sequencing with the use of a Nextera DNA Sample Preparation Kit (Epicenter). Index-tagged libraries were created, and 96 separated libraries were sequenced in each of eight channels using the HiSeq platform (Illumina) to generate 100 base pair (bp) paired-end reads. All sequences were submitted to the European Nucleotide Archive (ENA), and individual accession numbers are given in Table S1 (available in the online Supplementary Material). Sequence reads were mapped to reference genome HO 5096 0412 [10, 15] (GenBank reference number HE681097.1) using SMALT v0.7.4 [24] to identify single-nucleotide polymorphisms (SNPs). SNPs were filtered to remove those at sites with a SNP quality score below 30, and SNPs at sites with heterogeneous mappings were filtered out if the SNP was present in less than 75 % of reads at that site. Mobile genetic elements, phage and repetitive regions were excluded from the alignment, leaving a core genome size of 2 655 809 bp (A full list of excluded regions is available in Table S2). Phylogenetic trees were reconstructed using RaxML with the general time reversal model and gamma correction [25]. Twenty sequences belonging to sporadic community-acquired MRSA (with an SCCmec type other than type IVh) which are more diverse and do not belong to the EMRSA-15 linage were excluded from the analysis, leaving 1085 sequences.

### Patient referral network

We used data from the NHS Hospital Episode Statistics to reconstruct the patient referral network. Data about hospital admissions between April 2006 and March 2009 were analysed at patient level. The time frame for patient admission data was restricted to three years to limit the influence of changes in the health care system, such as mergers of hospitals. We thus assume that the observed three-year-based network is representative for the majority of patient movements spanning the sampling interval of the strain collection. For all patients, all consecutive admissions *A_ij_* to hospital *i* after a previous admission to hospital *j* were counted. This included both referrals that were direct from one hospital to another as well as indirect, after a stay at home. All patient readmissions were combined *m_ij_*=∑*A_ij_* in one matrix M={*m_ij_*}, which defines the weight of connectedness between any two hospitals based on the number of patients that they shared. We used a community assignment algorithm maximising modularity Q [26] to assign health care regions in the patient referral network, as described previously [5].

### Genetic population structure

In order to estimate the genetic flow between bacterial populations, we utilised an adapted version of the Wright's F statistic. We treated every nucleotide position as a separate gene, with the four nucleotides of the genetic code as the total number of possible alleles. For each position *i*, we calculated the percentage of each nucleotide in the sequences from the population *j* in question, *p_jix_*=*N_jix_*/*N_j_*, *x*={A,T,C,G}, where *N_j_* denotes the number of sequences from population *j* and N*_jix_* denotes the number of sequences with nucleotide *x* at position *i*.

The mean number of SNP differences between the sequences of a single population is a poor measure of the variation within the population when the number of single occurring SNPs (singletons) is relatively high. In our data 19 785 out of 26 337 SNP positions (75 %) were singletons. We therefore calculated for each nucleotide position the chance of picking two sequences from both populations, forming the same heterozygous pair. The underlying idea is that any SNP that is not completely present or absent from both populations can be informative about the exchange of bacteria between these populations. If we assume that each SNP only arises once, a SNP partially present in two populations is the result of an introduction into one or both of the populations.

Only a small number of nucleotide positions [22 (0.3 %), excluding the singletons] contained more than two variants and given each SNP location i the sum over the probabilities of picking two heterozygous SNP pairs (one pair from population 1, with proportion *p*_1*i*_, and one pair from population 2, with proportion *p*_2*i*_) can therefore be written as

$$H_{p}=\sum_{i}p_{1i}\left(1-p_{1i}\right)p_{2i}\left(1-p_{2i}\right),$$

denoting the between-population variation. Within each of the populations, the sum over the probabilities of picking two identical heterozygous pairs then defines *H_Sj_*=Σ*_i_* (*p_ji_*(1-*p_ji_*))^2^ and the mean over the two populations H_S_=(H_S1_+H_S2_)/2 then denotes the within-population variation. Our *F_SP_* is then calculated as

$$F_{sp}=(H_{s}-H_{p})/H_{s}=1-HP/HS,$$

which ranges between 0 (*H_P_*=*H_S_*) and 1 (*H_P_*=0).

In order to calculate the divergence of a single population, *h*, we calculated *F_ST_* in the same way as *F_SP_*, except we assumed the rest of the populations to be one single joined population. This is done by taking the mean over all SNP frequencies for the other populations, *p*_2*ix*_=Σ_*k*≠*h*_*p_kix_*/(*N_k_*−1), where *N_k_* is the number of populations.

### Population differences

Isolates were grouped into populations by hospital, and any population with fewer than five isolates was excluded. To allow the ascertainment of temporal differences in bacterial populations, we split each hospital's population into time periods of three years (1998–2000, 2001–2003, 2004–2006, and 2007–2009, and 2010–2012) in a parallel analysis, again excluding all populations with fewer than five isolates. For regions and countries, we assumed SNP frequencies per hospital were representative for the populations and calculated the SNP frequencies for each population at these levels as the mean SNP frequencies over the included hospitals.

To test if the measured divergence of populations deviated from what could be expected at random given the structure of our data, we performed permutation tests based on different levels of isolate grouping. In the first permutations, we randomised all isolates, keeping the number of isolates per hospital equal to the original dataset. In the parallel permutation, we randomised the hospitals, keeping their number per region and country constant. For each level we created 1000 permutations and calculated *F_ST_* for all populations in each dataset.

We calculated the *F_SP_* for each pair of regions and compared these with random permutations of all hospitals in both regions disregarding the original geographical partition. These permutations were repeated 1000 times. Furthermore, we calculated *F_SP_* between all possible pairs of hospital-time frames for which more than five isolates were available.

In order to test the effect of inter-connectedness between hospitals within each health care region on the bacterial genetic population structure, the data from all hospitals in each region containing three or more hospitals were combined. When combining the data from multiple hospitals, for instance into regions, we assumed that the samples from one hospital are representative for the entire MRSA population of that hospital, and took the mean of the SNP proportions of the hospitals as the proportions for the combined set, *p_Rix_*=∑ _*j*∈*r*_*p_jix_*/*N_R_*, where *N_R_* denotes the number of hospitals in region R.

### Travel time between hospitals

The geographical position of the hospitals was determined using their postcodes, and distances between all hospitals was measured as the travel time by car, in absence of traffic, between the geo-locations using the Open Source Routing Machine, based on the OpenStreetMap database (http://project-osrm.org).

### Possible introductions

To identify introduction events, we selected sub-clades with five or more isolates from the phylogeny. We consider a possible introduction (PI) to be represented by an isolate assigned to an otherwise geographically homogeneous clade (all isolates from the same hospital) that was isolated from a patient in another hospital. Monophyletic isolates from the same destination hospital were counted as a single PI. We took a conservative approach and only assigned PIs where the putative donor hospital was represented by at least four isolates and contributed at least 80 % of the isolates within the clade.

## Results

**Fig. 1.**
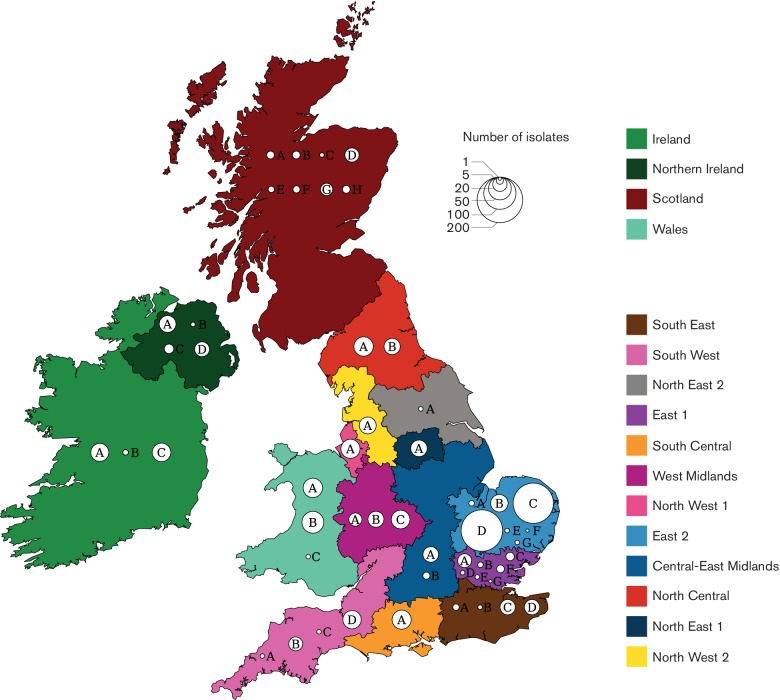
Overview of hospitals, health care regions and MRSA samples. The map of the United Kingdom and Ireland, coloured by referral cluster, depicting Wales, Scotland, Northern Ireland, and the Republic of Ireland indicated as single referral clusters. Each circle represents a hospital, identified by a letter unique to the region, on an arbitrary place in the region. Circle sizes represent the number of isolates included per hospital.

The total collection comprised 1085 CC22 isolates, of which 1074 isolates were EMRSA-15 (ST22), originating from 52 hospitals across the United Kingdom and the Republic of Ireland (henceforth UK and I, Fig. 1a). Thirty-three hospitals submitted five or more isolates. Two hospitals in region East-2 (Hospital C and Hospital D) were over-represented in our dataset due to in-depth sampling, with 157 and 166 isolates, respectively. Hospitals in England were divided according to 12 ‘referral regions’ based on the structure of the patient movements between all hospitals (Table S3) as previously described [21, 27]. Three regions included three hospitals with five or more isolates (East-1, East-2 and West Midlands), and one region lacked any hospital with enough isolates (North-East 2).

**Fig. 2.**
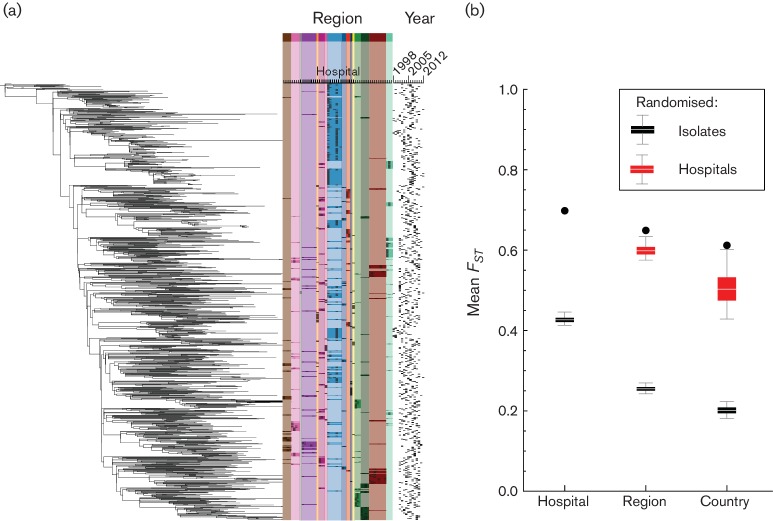
(a) The MRSA CC22 phylogeny, including all 1085 isolates, shows clear regional clustering (coloured bands), with some clustering at the hospital level, while there is little or no temporal clustering of isolates. A larger version of the phylogeny can be found in Fig. S1. (b) The mean *F_ST_* (Black dots) for each level of the health care system differs significantly from permuted datasets [Box-whiskers, with central lines showing median, boxes showing inter-quartile range, and whiskers showing the 95 % (2.5–97.5 %) interval].

The phylogeny based on the 1085 whole-genome sequences showed clear regional clustering (Fig. 2a), exemplified by the large clusters from Northern Ireland (note the cluster at the bottom of the tree in Fig. 2a). Isolates from single hospitals also clustered, which was particularly apparent for the over-represented hospitals in region East-2. In contrast little or no temporal clustering was evident, thus bacterial populations within a single region seem to remain relatively stable over time.

In order to quantify the genetic similarity between bacterial populations we used Wright's F-statistic, which is a standard population genetics metric commonly used to estimate the degree of gene flow, where a value of 0 indicates that populations are identical, and a value of 1 indicates that populations are completely isolated from each other. We first calculated the mean *F_ST_*, comparing single populations with the entire combined population, over the different levels of the health care system: hospitals, referral regions (as defined by the structure of the patient referral network), and countries. The mean *F_ST_* for each level was higher than would be expected if isolates were distributed randomly (Fig. 2b), indicating that isolates within the bacterial populations at each level were generally more related to each other than those in other bacterial populations at that level. When randomising the bacterial populations of hospitals, instead of single isolates, over the available hospital locations, we found that the mean *F_ST_* for regions and countries was also higher compared with the permutations randomising hospital locations.

**Fig. 3.**
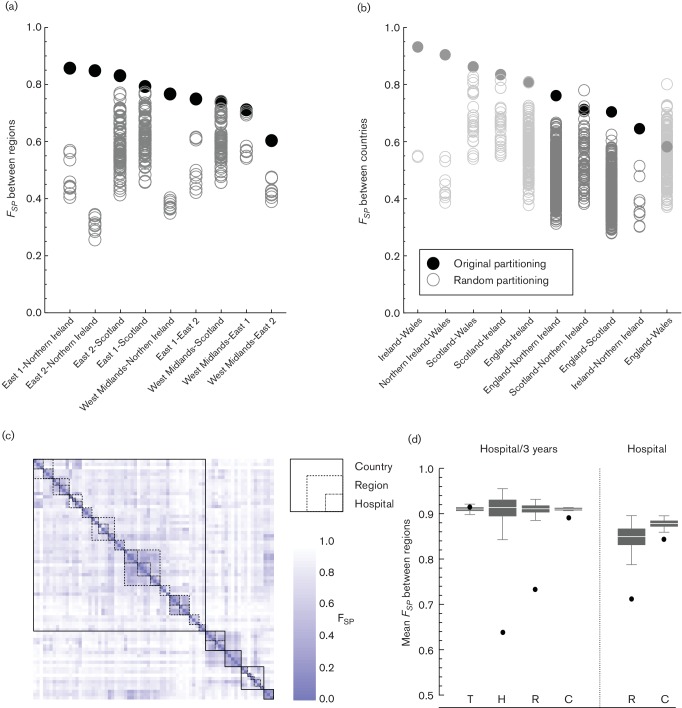
Comparing hospital populations using Wright’s F-statistic. (a) The partitioning of hospitals according to their referral region delivers the best distinction between genetic populations. The *F_SP_* values of the genetic populations between any two regions are significantly higher when using the original partitioning compared with any other possible partitioning of those hospitals. (b) On the level of countries, while excluding countries with fewer than three hospitals available (in grey), just two possible divisions results in higher *F_SP_* than the original partitioning (both between Northern Ireland and Scotland). (c) The distance matrix shows the *F_SP_* value between each of the hospitals, divided into 3-year intervals. Boxes show the *F_SP_* values that fall within single hospitals, regions and countries. (d) The mean *F_SP_* values within each level (H, Hospital; R, Region; C, Country) are lower than expected at random. This applies to both *F_SP_* values between hospital-time-frames and between hospitals. The distances between time-frames (T) from the same years is not different.

The original partitioning of hospitals in referral regions fitted better (i.e. maximised the pair-wise F-statistic, *F_SP_*, between regions) than most of the random partitions (Fig. 3a, b). Even for East-1 and East-2, the only referral regions in this analysis with a border and with three or more hospitals in the dataset, the bacterial populations differed significantly from the random partitions, despite the geographical proximity. In only two instances did a random partitioning at the country level result in a higher *F_SP_*. Both involved a partitioning between Scotland and Northern Ireland that included hospitals A, E and G in Scotland (all in the West of Scotland) and either hospital Scotland-D (*F_SP_*=0.780) or hospital Scotland-B (0.723) being included with the hospitals in Northern Ireland, instead of with the rest of Scotland (0.714). Otherwise health care regions defined on the basis of the observed patient referral between hospitals were the best way to divide the bacterial populations of all hospitals.

The genetic differences between pairs of bacterial populations show the clear hierarchical structure of the MRSA population in the UK and I (Fig. 3c). Bacterial populations within each hospital were genetically most similar, followed by those within each region, and finally those within each country (Fig. 3d). This was also observed when comparing the bacterial populations of hospitals irrespective of the time of isolation. Bacterial populations from the same time period from different hospitals as equally divergent as any randomly chosen combination of populations, indicating that the MRSA populations within hospitals are largely conserved over time.

**Fig. 4.**
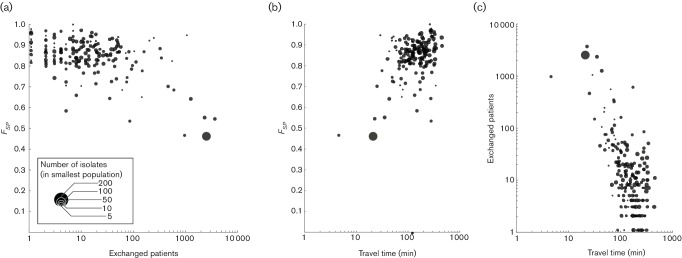
The *F_SP_* between all pairs of available hospitals in England as a function of distance between them, with circle size denoting the size of the smallest population size of the two. (a) Expressing distance as the number of patients exchanged between them on a yearly basis. With increasing number of exchanged patients, the hospitals’ populations become genetically more similar. (b) Hospitals in close geographical proximity, expressed in travel time by car between them, have genetically more similar MRSA populations. (c) Patient transfers have a strong geographical component, with more patients being transferred between hospitals in close geographical proximity.

Hospitals that shared more patients generally showed genetically more similar bacterial populations (Fig. 4a, Mantel test *P*<0.001). This was particularly apparent for hospitals that exchanged more than 100 patients per year, after which *F_SP_* values dropped considerably. Hospitals with larger sample sizes showed a consistent dependency on patient exchanges whereas those with fewer isolates showed a larger variability in *F_SP_*. However, the effect of patient exchanges could not be directly distinguished from the effect of geographical distance between hospitals (measured in travel time, Fig. 4b, Mantel test *P*<0.001), owing to the strong correlation between the two (Fig. 4c, Mantel test *P*<0.001).

**Fig. 5.**
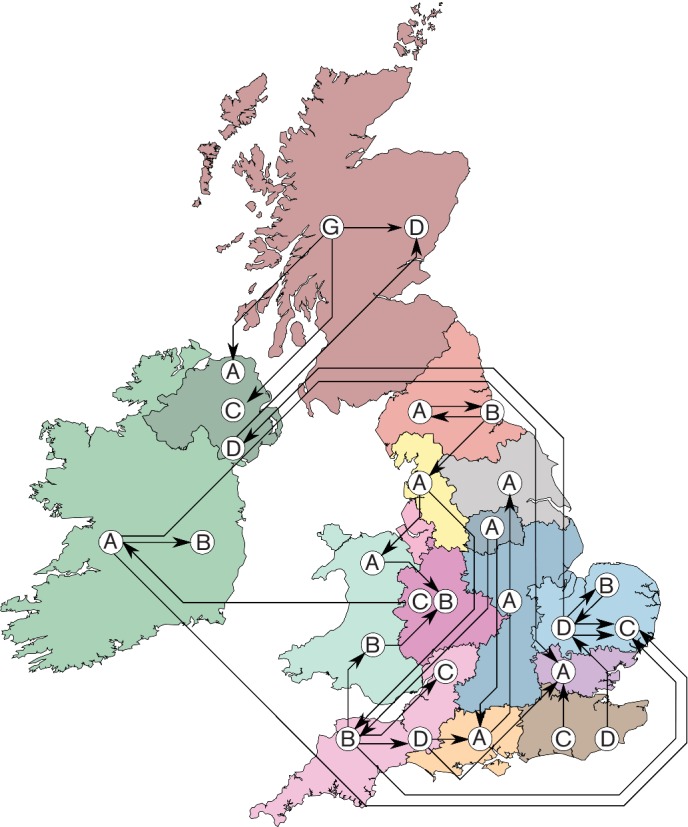
Possible introductions of MRSA between hospital populations can be inferred from the phylogeny. Arrows show introductions between hospitals (Indicated by letters per region), from the predominant hospital of the clade to the hospital that submitted the discordant isolate. While most possible introductions occur within the same region or between neighbouring regions, a number of introductions span considerable distances.

Among all isolates, we identified 30 that mapped to a clade occupied by a majority from another single hospital. These isolates thus show the hallmarks for introductions from one hospital to another. Although this introduction could have happened indirectly, through an intermediate health care institution, the donor hospital's bacterial population was the most likely source. Many of these introductions occurred at relatively short distances, yet a small number of introductions occurred over considerable distances (Fig. 5).

## Discussion

By sequencing a large, representative collection of the most prevalent MRSA clonal complex throughout the UK and the Republic of Ireland, we were able to give a comprehensive description of its population structure, and propose its most likely dispersal dynamics. Our results indicate that the spread of MRSA is governed by a hierarchy of processes acting simultaneously on multiple levels – within single hospitals, between hospitals within health care regions, between regions within a country and between countries, that reflect the structure of patient movements throughout the health care system. The identification of patient movements as a potential transmission pathway is important to the design of effective control strategies to reduce the transmission of MRSA and other antibiotic-resistant micro-organisms between patients and hospitals.

The analysis provides clues as to the most apposite geographical and temporal scales with which to describe the distribution of MRSA variants. A large proportion of the expansion of MRSA took place within single hospitals, resulting in the population of given hospital in a given year being more similar to the population of the same hospital in other years than to those of other contemporaneous hospital populations. This indicates that the population of MRSA circulating within each hospital is stable over time, and is not frequently perturbed by introductions from other hospitals. It does not necessarily mean, however, that there was a continuous transmission within the hospital at all times, and the role of community reservoirs within the hospital catchment area also needs to be considered. Patients who were MRSA-colonised during one hospital stay might reintroduce the organism during subsequent stays. The observation that sustained MRSA populations exist in single hospitals does, however, justify continued emphasis on strong infection control within hospitals.

The bacterial populations within hospitals are not completely isolated, as the association between bacterial populations extends from the same hospital to hospitals in the same health care region, apparent as a coherent regional genetic population structure. There were only two instances where a random partition of hospitals provided a better fit of the observed genetic diversity than the geographical division. Both cases concerned hospitals in Scotland and Northern Ireland, areas in which the true patient referral pattern was not included. It is likely that hospitals in the East and West of Scotland should be treated as different health care regions based on patient movements [28, 29], and not be assigned arbitrarily as single entity along administrative borders as was done in the present analysis.

Bacterial populations of different countries are largely segregated, in particular between Ireland (including Northern Ireland) on the one hand and England, Scotland and Wales on the other. This country-specific clustering is likely to reflect the observation that patients are less likely to travel across national borders to seek healthcare unless there is a very good reason to do so. Furthermore, this finding is consistent with previously observed country-specific clades in the MRSA ST-22 population structure [15] and the clustering of different clonal complexes observed in other European countries [13]. The reduced flow of patients across countries' borders thus seems to mitigate the international dispersal of MRSA and other health care-associated infections. This should be taken into account when considering the likely increase in patients seeking health care across borders, facilitated by changes in policy (such as EU directive 2011/24/EU, that stipulates the right of EU citizens to seek health care in other EU countries).

Our findings confirm that hospitals that exchange many patients had genetically more similar bacterial populations than hospitals further afield, as found by other studies [19, 20]. This process is likely to be driven by the exchange of MRSA colonised or infected patients between hospitals. However, we could not distinguish spread through the general human population from spread through hospitals by transferred patients. This is caused by the strong geographical component in patient transfers, as patients and doctors are likely to choose the closest hospital that offers the required treatment. However, dispersal through patient movements seems more likely, given the agreement between the division of the patient referral network in referral regions and the genetic population structure of MRSA in the hospitals. Irrespective of the exact mode of transmission between hospitals, the proximity of hospitals influences the chances of inter-institutional dispersal. Control strategies should take this inter-hospital dispersal into account, for instance through regional coordination of infection prevention and control efforts, as the failure of control in one hospital will impinge on its surrounding health care institutions [30, 31].

We were also able to describe a limited number of possible introductions of MRSA over medium- and long-range distances. Although the underlying process for these introductions remains elusive, and the international transfer, and subsequent establishment, of MRSA appear to be relatively rare events [32, 33], it does illustrate that regions and countries are not completely isolated entities and experience a constant pressure of introduction. One possible explanation for these introductions is the necessity for patients to travel for highly specialised treatments (e.g. transplantation), bringing with them a strain prevalent in their home hospital to the far away hospital or vice versa. However, these long-distance exchanges may also have other causes, such as the movement of health care workers, or patients’ personal residential choices.

### Limitations

We acknowledge several limitations of our study. First, our sample was largely based on the isolates collected for the BSAC Bacteraemia Resistance Surveillance Programme [22, 23], and as such includes only a proportion of all hospitals in the UK and the Republic of Ireland and a small proportion of all MRSA-positive samples within each hospital. Among the included hospitals, some submitted very few MRSA isolates, and as such were excluded from part of the analysis. The unobserved hospitals' bacterial populations may have influenced some of the results. In particular, some of the included isolates may have been imported from unaccounted hospitals, linking hospitals through unobserved bacterial populations. Furthermore, this sampling scheme greatly reduces the number of hospital pairs in the network that exchange a high number of patients (>1000 patients/year), making the identification of mixed bacterial populations more difficult.

To make sure that we observed hospital pairs that exchanged a high number of patients, we included isolates from hospitals in the east of England. This resulted in both a higher number of hospitals, as well as a higher number of isolates, from region East-2 in particular. This oversampling may have resulted in more possible introductions being identified to and from this region but is unlikely to make a large difference for the other results and final conclusions.

To test if the genetic composition of the hospital MRSA populations were consistent with the regional structure of the patient referral network, we used a permutation test that was restricted to the original size partitioning of the regions. It did not test all possible divisions of the available hospitals, which is next to impossible given the large number of possible partitions. However, by using this pair-wise approach we expect to have tested the most meaningful partitions, combining hospitals that can be expected to present genetically similar bacterial populations. Furthermore, we defined these health care regions using a community assignment algorithm that maximises modularity Q [26]. This modularity reflects the number of exchanged patients within regions relative to the exchanges between regions. It is not guaranteed that this delivers the most meaningful partitioning reflecting the dispersal of MRSA. The South-East and East-1 regions, for instance, exchange 10 times as many patients between them than two typical other regions, which may in turn cause more introductions of MRSA between these regions. Although other methods for finding community structure in networks are available [34], the relative advantages and limitations of these different methods relating to the population structure of a pathogen spreading through the network remain unclear.

Lastly, a combined UK patient referral network is difficult to construct because patient referral data are not directly comparable between countries. Northern Ireland and the Republic of Ireland had no patient-level data available, while coupling the Scottish patient data with the English and Welsh would probably have led to patients not being linked during cross-border exchanges, confounding the network. We therefore used only patient movement data from England, and defined the other countries as single health care regions. As previously discussed this affected the results for Scotland in particular, emphasising the importance of the analysis of the patient referral networks. Despite these limitations, however, we were able to disentangle the genetic population structure of MRSA using the structure of patient referral patterns.

### Conclusions

The control of healthcare-associated infections caused by antibiotic-resistant organisms is currently the responsibility of individual hospitals. As a consequence, public health policy and infection control measures are targeted towards individual hospitals, rather than the wider healthcare system. Our data indicate that there is a high level of temporal stability of the bacterial population circulating within any given hospital, suggesting that a large part of the dispersal and expansion of MRSA takes place among patients seeking care in single hospitals. However, this individual hospital approach may only partially be justified, as it does not take into account the potential influence of other hospitals.

Inter-hospital spread of resistant bacteria is by no means a rare occurrence; our results show how hospitals are exposed to constant introductions of MRSA on a number of levels, from single hospitals, through regions to entire countries. We propose that patient transfers between hospitals are the likely process behind the bacterial exchanges, although spread through the general population, by movement of health care workers or other mechanisms cannot be ruled out. This may however depend on the nature of the bacterial lineages in question, with community-associated strains relying less on patient transfers. Nevertheless, the hierarchical nature of the MRSA population structure mirrors the levels of patient sharing, with most MRSA received from hospitals that directly transfer large numbers of patients. Fewer introductions happen between health care regions or across national borders, reflecting lower numbers of transferred patients. Even over these long distances, however, multiple introductions were identified.

Knowledge of the inter-hospital dispersal of MRSA and other health care-associated pathogens is crucial for the design of effective control strategies. The exposure to MRSA between hospitals imposes a shared responsibility for infection control efforts. Our results indicate that these control efforts are best coordinated regionally, among groups of hospitals that share patients. It is worth emphasising that these regions are determined by the movements of patients between hospitals, and do not necessarily overlap with the administrative regions of the health care system. The absence of a collaborative design of infection control measures can give the bacteria the opportunity to escape to other hospitals, undermining control efforts. A joint coordinated control effort between hospitals, at the very least at a regional level, is paramount for the national control of MRSA and other hospital-associated pathogens.

We also demonstrated the usefulness of whole-genome sequencing (WGS), for determining the relatedness of bacterial populations from different hospitals and regions, where conventional typing methods would have failed. As WGS becomes more widely available and sequence databases that provide epidemiological information are bound to grow, our understanding about the dynamics of transmission and the dispersal of bacteria and the role of reservoirs will undoubtedly inform better and more targeted intervention strategies. With the incorporation of WGS as part of the routine diagnostic procedures surveillance systems can tap into this rich resource.

## Data bibliography

1. Donker T, Reuter S, Sciberas J, Reynolds R, Brown N *et al.* MRSA diversity project collection; European Nucleotide Archive project accession number PRJEB2655. http://www.ebi.ac.uk/ena/data/view/PRJEB2755 (2017).
2. Donker T, Reuter S, Sciberas J, Reynolds R, Brown N *et al.* British Society for Antimicrobial Chemotherapy (BSAC) collection; European Nucleotide Archive project accession number PRJEB2756. http://www.ebi.ac.uk/ena/data/view/PRJEB2756 (2017).
3. Donker T, Reuter S, Sciberas J, Reynolds R, Brown N *et al.* East of England collection; European Nucleotide Archive project accession number PRJEB2944. http://www.ebi.ac.uk/ena/data/view/PRJEB2944 (2017).

## Supplementary Data

66 Supplementary File 1

## Supplementary Data

66 Supplementary File 2

## Supplementary Data

66 Supplementary File 3

## Supplementary Data

66 Supplementary File 4
