# Supplementary figures and images for: Population genetic structuring of methicillin-resistant Staphylococcus aureus clone EMRSA-15 within UK reflects patient referral patterns
Source: Microb Genom. 2017 Jul 4;3(7):e000113. doi: 10.1099/mgen.0.000113 (PMC5605955; doi:10.1099/mgen.0.000113)

Region

Year

Hospital

1998  
2005  
2012

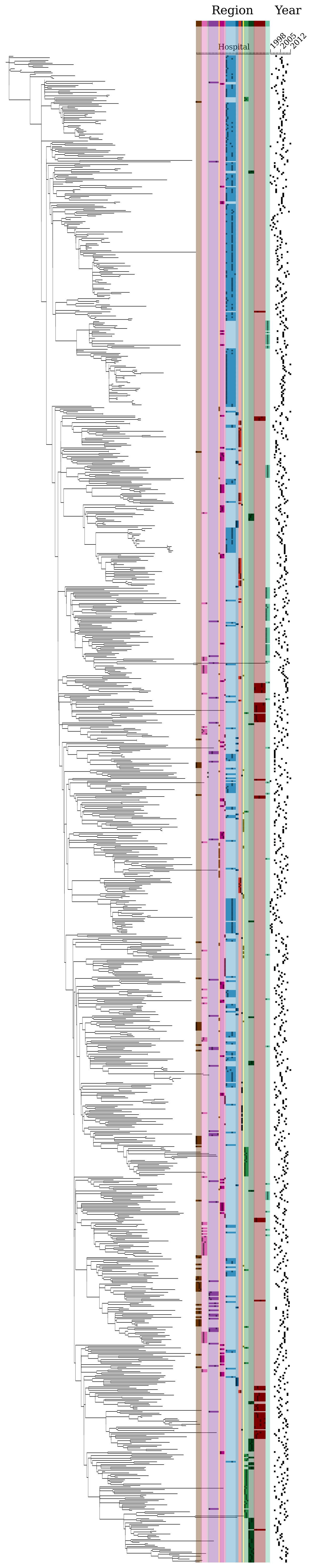

Supplement: Supplementary File 1 [file mgen-3-113-s001.pdf]
