# Supplementary material for: Population genetic structuring of methicillin-resistant Staphylococcus aureus clone EMRSA-15 within UK reflects patient referral patterns
Source: Microb Genom. 2017 Jul 4;3(7):e000113. doi: 10.1099/mgen.0.000113 (PMC5605955; doi:10.1099/mgen.0.000113)
Supplement: Supplementary File 3 [file mgen-3-113-s003.pdf]

Position	Description
34163 – 51525	Staphylococcal cassette chromosome mec (SCCmec) type IV
80765 – 83026	IS element
137322 – 138041	IS element
141474 – 142274	IS element
315945 – 318143	IS element
581228 – 581800	repeat region
585722 – 586297	repeat region
589680 – 590150	repeat region
818385 – 820574	IS element
824723 – 825556	repeat region
935317 – 936294	IS element
938088 – 938798	IS element
977393 – 979583	IS element
1104852 – 1107038	IS element
1252874 – 1254311	IS element
1283817 – 1286016	IS element
1328804 – 1330543	IS element
1361018 – 1375548	ICE-like element
1520143 – 1566314	phiSLT and phi PVL like phage
1567574 – 1569770	IS element
1852864 – 1853851	IS element
1861820 – 1863920	duplication
1874756 – 1876610	IS element
1890687 – 1892984	IS element
1943176 – 1944547	IS element
1944548 – 1945123	IS element
2098579 – 2099979	putative phage protein
2021753 – 2029411	Tn552-like
2042948 – 2087965	phiSa3 Prophage
2103705 – 2103914	repeat region
2136387 – 2138610	IS element
2407746 – 2408446	repeat region
2509256 – 2510560	IS element
2568107 – 2570285	IS element
2700680 – 2701410	IS element
2737370 – 2738209	repeat region
2776273 – 2778047	repeat-rich region
2825393 – 2826315	IS element
